# Pathogenesis of lassa fever in cynomolgus macaques

**DOI:** 10.1186/1743-422X-8-205

**Published:** 2011-05-06

**Authors:** Lisa E Hensley, Mark A Smith, Joan B Geisbert, Elizabeth A Fritz, Kathleen M Daddario-DiCaprio, Tom Larsen, Thomas W Geisbert

**Affiliations:** 1 Virology, US Army Medical Research Institute of Infectious Diseases, Fort Detrick, MD, USA; 2 Pathology Divisions, US Army Medical Research Institute of Infectious Diseases, Fort Detrick, MD, USA; 3 Galveston National Laboratory, University of Texas Medical Branch, Galveston, TX, USA; 4 Department of Microbiology and Immunology, University of Texas Medical Branch, Galveston, TX, USA

## Abstract

**Background:**

Lassa virus (LASV) infection causes an acute and sometimes fatal hemorrhagic disease in humans and nonhuman primates; however, little is known about the development of Lassa fever. Here, we performed a pilot study to begin to understand the progression of LASV infection in nonhuman primates.

**Methods:**

Six cynomolgus monkeys were experimentally infected with LASV. Tissues from three animals were examined at an early- to mid-stage of disease and compared with tissues from three animals collected at terminal stages of disease.

**Results:**

Dendritic cells were identified as a prominent target of LASV infection in a variety of tissues in all animals at day 7 while Kupffer cells, hepatocytes, adrenal cortical cells, and endothelial cells were more frequently infected with LASV in tissues of terminal animals (days 13.5-17). Meningoencephalitis and neuronal necrosis were noteworthy findings in terminal animals. Evidence of coagulopathy was noted; however, the degree of fibrin deposition in tissues was less prominent than has been reported in other viral hemorrhagic fevers.

**Conclusion:**

The sequence of pathogenic events identified in this study begins to shed light on the development of disease processes during Lassa fever and also may provide new targets for rational prophylactic and chemotherapeutic interventions.

## Introduction

Lassa fever (LF) is a severe hemorrhagic fever (HF) that is endemic in West African countries with the highest incidence in the Republic of Guinea, Sierra Leone, Liberia, and Nigeria. It is estimated that Lassa virus (LASV) infects over 300,000 individuals per year across this region, causing over 3,000 deaths [1]. The case fatality rate for LF is estimated to be approximately 15% in hospitalized patients and has been reported to be greater than 50% in several outbreaks [1,2]. Human infection is associated with contact with a widely distributed and highly commensal rodent, *Mastomys natalensis*, or by contact with infected patients. Recent importation of LF into Germany, the Netherlands, the United Kingdom, and the United States by travelers on commercial airlines [3-9] illustrates the potential for the spread of this highly dangerous and contagious pathogen. In addition, LASV has gained notoriety because it is classified as a Category A bioweapon agent [10].

Currently, there are no vaccines or antiviral drugs approved for LF. Treatment with intravenous ribavirin was shown to reduce mortality from LF in high-risk patients and presumably decreases morbidity in all patients with LF [11,12]. However, the availability of ribavirin is very limited in endemic areas and the effectiveness of treatment is limited if not initiated within the first week of disease onset [11,12]. Preventing contact with the reservoir host in endemic areas is currently unachievable. Because adequate and well-controlled clinical studies in humans cannot be ethically conducted and as field efficacy studies are not yet feasible for LF, licensure of any of these countermeasures must be carried out under the U.S. Food and Drug Administration (FDA) "Animal Efficacy Rule. This policy was implemented in 2002 and allows for the evaluation of vaccines or therapeutics using data derived from studies carried out in an animal model that accurately reflects the disease observed in humans. Importantly, the animal model must be well-characterized and the pathophysiology of the disease in the animal must faithfully reproduce disease in humans.

Currently, there is no immunocompetent mouse model for LASV. Strain 13 guinea pigs are susceptible to a strain of LASV (Josiah) from Sierre Leone with uniform mortality 15-19 days following i.m. inoculation [13]. While guinea pigs are useful for screening vaccines and antiviral drugs the disease caused by LASV infection does not faithfully reproduce the human condition [14]. Several primate species have been evaluated as potential models for LF including squirrel monkeys, capuchin monkeys, marmosets, hamadryas baboons, African green monkeys, cynomolgus monkeys and rhesus monkeys [15-34]. While capuchin and squirrel monkeys seroconverted after LASV challenge both species were uniformly resistant to lethal disease [14,16]. Interestingly, low challenge doses (10-15 pfu) of the Josiah strain of LASV produce uniformly lethal disease in African green monkeys and rhesus macaques whereas high challenge doses (1.2 × 10^6 pfu) produce only partially lethal infections [14]. This same phenomenon was not observed in cynomolgus macaques [14] which appear to be more susceptible to lethal LASV infection than these other species.

Most studies have used cynomolgus or rhesus monkeys as a model to evaluate vaccines and/or treatments against LF [17,18,21,22,27,29-31], while several studies have evaluated the pathogenesis of LF infection in macaques [19,20,23-25,34]. However, with few exceptions, previous investigations examined tissues of infected animals euthanized when moribund, and shed little light on the pathogenesis of LF infection during times before death. The aim of this pilot study was to begin to characterize the earlier stages of LF in a relevant nonhuman primate model of human disease.

## Materials and methods

### Animals and inoculations

Six healthy, LASV-seronegative, cynomolgus (*Macaca fascicularis*) macaques (3 to 4 kg) were used for these studies. Animals were inoculated in the caudal thigh with a calculated dose of 3000 plaque forming units (PFU) of LASV (Josiah strain). Longitudinal blood samples were collected before challenge and on days 3, 6 (herein referred to as the actual closer time of 5.5 days), 10, and 14 (herein referred to as the actual closer time of 13.5 days) after challenge and analyzed by complete blood counts, clinical chemistry, coagulation assays, and fluorescence-activated cells sorter analysis of various cell populations (described below). In addition, blood samples were collected at the time of euthanasia. Each monkey was specifically evaluated for anorexia, diarrhea, nasal exudates, vomiting, conjunctivitis, cutaneous rash, dehydration, central nervous system disturbances, reduced activity, and hemorrhage using a subjective scoring system. Three animals were euthanized on day 7 after LASV challenge while the remaining three animals were euthanized when clinical signs indicated terminal disease according to an endpoint scoring sheet.

### Hematology

Total white blood cell counts, white blood cell differentials, red blood cell counts, platelet counts, hematocrit values, total hemoglobin, mean cell volume, mean corpuscular volume, and mean corpuscular hemoglobin concentration were determined from blood samples collected in tubes containing EDTA, using a laser-based hematologic analyzer (Coulter Electronics, Hialeah, FL).

### Serum biochemistry and coagulation assays

Concentrations of albumin (ALB), amylase (AMY), alanine aminotransferase (ALT), aspartate aminotransferase (AST), alkaline phosphatase (ALP), gamma-glutamyltransferase (GGT), glucose (GLU), cholesterol (CHOL), total protein (TP), total bilirubin (TBIL), urea nitrogen (BUN), and creatinine (Cr) were measured using a Piccolo Point-Of-Care Blood Analyzer (Abaxis, Sunnyvale, CA). Plasma levels of fibrin degradation products (D-dimers) were quantitated by an enzyme-linked immunosorbent assay (ELISA) according to manufacturer's directions ("Asserachrom D-D", Diagnostica Stago, Inc., Parsippany, NJ). Plasma levels of protein C were determined by a chromatic hydrolysis assay (DiaPharma, West Chester, OH) as described elsewhere [35].

### Cytokine/chemokine production

Cytokine/chemokine levels in monkey plasma were assayed using a human cytokine multiplex-25 bead array assay kit (BioSource, Camarillo, CA) for the Bio-Plex 200 System (Bio-Rad, Hercules, CA) according to manufacturer's directions. Cytokines/chemokines assayed included eotaxin, granulocyte-macrophage colony-stimulating factor (GM-CSF), interferon (IFN)-α, IFN-γ, IL-1β, IL-1 receptor antagonist (IL-1RA), IL-2, IL-2R, IL-4, IL-5, IL-6, IL-7, IL-8, IL-10, IL-12p40/p70, IL-13, IL-15, IL-17, IFN-γ-inducing protein (IP)-10, monocyte chemoattractant protein (MCP)-1, macrophage inflammatory protein (MIP)-1α, MIP-1β, monokine induced by γ-IFN (MIG), regulated upon activation normal T-cell expressed and secreted (RANTES), and tumor necrosis factor (TNF)-α.

### Histology

A complete necropsy was performed on all animals. Tissues were collected and immersion-fixed in 10% neutral-buffered formalin and processed for histopathology and phosphotungstic acid hematoxylin (PTAH) staining to demonstrate polymerized fibrin as previously described [35].

### Immunohistochemistry

Tissue sections were deparaffinized and rehydrated through a series of graded ethanols, pretreated with DAKO Ready to Use Proteinase K (DAKO, Carpinteria, CA) for 6 minutes at room temperature, and blocked with DAKO's Serum Free Protein Block for 20 minutes before exposure to antibody. Sections were incubated with primary antibody overnight a 4°C using a mouse monoclonal antibody against LASV GP2 (USAMRIID, L52-121-22-BA02) (1:4000). Sections were then exposed to a peroxidase- or alkaline phosphatase-labeled polymer (Envision™, DAKO Corp., Carpenteria, CA) for 30 minutes. Color development was attained by exposing tissue to one of three substrate-chromagens, either 3,3'-diaminobenzidine (DAB) for 5 min; or 6-bromo-2-hydroxyl-3-naphtholic acid (Histomark Red, Kirkegaard and Perry, Gaithersburg, MD) for 50 minutes in the dark; or DAKO Permanent Red for 50 minutes in the dark. Sections were counter-stained with hematoxylin. Negative controls included replicate sections exposed to anti-LASV virus antibodies and unexposed cynomolgus monkey tissue; archived LASV-infected cynomolgus tissue served as positive controls.

### Terminal Deoxynucleotidyl Transferase-Mediated Deoxyuridine Triphosphate Nick End Labeling (TUNEL) staining

The ApopTag detection kit (S7100 kit; Chemicon, Billerica, MA) was used to detect cells undergoing apoptosis in the spleen, lymph nodes, and liver. Sections were stained in accordance with kit instructions with the following modifications; a 13-minute room temperature protein digestion in prediluted proteinase K (DAKO) was used. Slides were incubated in equilibration buffer for 5 minutes, followed by incubation in working strength terminal deoxynucleotidyl transferase (TdT) enzyme at 37°C for 60 minutes. This was followed by a stop/wash step and an anti-digoxigenin peroxidase step, which were identical to kit instructions. Color development was with DAB chromagen and then slides were counterstained with hematoxylin.

### Virus isolation

Infectious virus in EDTA plasma was assayed by counting plaques on Vero cells maintained as monolayers in 6-well plates under agarose, as previously described [31]. Portions of liver; spleen; lung; kidney; adrenal gland; pancreas; heart; ovary; brain; femoral bone marrow; and mandibular, mesenteric, axillary, and inguinal lymph nodes were aseptically collected during necropsy and stored at -70°C until assayed for infectious LASV. After thawing, tissues were weighed and ground by mortar and pestle with alundum in 5 ml of EMEM with Earl's salts with 10% fetal calf serum. Tissue homogenates were centrifuged at 10,000 × g for 15 minutes and viral titers were determined as detailed above.

### Statistical analysis

Student's *t *test was used to compare cytokine/chemokine values in pre-challenge versus post-challenge samples. Statistical significance was assumed when p < 0.05.

## Results

### Clinical illness

Clinical findings are presented in Table 1. The first signs of clinical illness were noticed at day 3 when four of the six (4/6) animals given physical examinations had fevers (defined as temperature over 103°F). Bleeding at the venipuncture site was noted in 1/3 animals at day 7 and 2/3 animals at day 10. Mild dehydration (approximately 5% as evaluated subjectively by skin-fold retraction) was observed in 3/3 animals by day 10. All three remaining animals on day 10 were anorexic and showed signs of mild to moderate depression. By day 13.5, clinical illness progressed with animals becoming more anorexic and dehydrated. One animal developed neurologic symptoms including convulsions as well as seizures which increased in length and severity; this animal was euthanized on day 13.5. Facial edema and epistaxis were noted in 1/2 remaining animals by days 15-17. The two remaining animals were euthanized at days 15 and 17, respectively, using a subjective scoring system.

**Table 1:** Clinical findings in cynomolgus monkeys infected with Lassa virus*

Days Postinfection	Fever	Rash	Bleeding	Anorexia	Dehydration	Recumbency
0	0/6	0/6	0/6	0/6	0/6	0/6
3	4/6	0/6	0/6	0/6	0/6	0/6
5.5	2/6	0/6	0/6	0/6	0/6	0/6
7	2/3	0/3	1/3	0/3	0/3	0/3
10	2/3	0/3	2/3	3/3	3/3	0/3
13	0/1	0/1	0/1	1/1	1/1	1/1
13.5	0/2	0/2	0/2	2/2	2/2	0/2
15	0/1	1/1	0/1	1/1	1/1	1/1
17	0/1	0/1	1/1	1/1	1/1	1/1

*Data are presented as the number of animals in which a clinical finding was observed followed by the total number of animals examined. Fever and dehydration were evaluated in animals anesthetized for phlebotomy and/or physical examination. Rash, bleeding, anorexia, and recumbency were monitored in all remaining animals at the specified time point.

### Hematology, clinical chemistry, and coagulation

**Figure 1:**
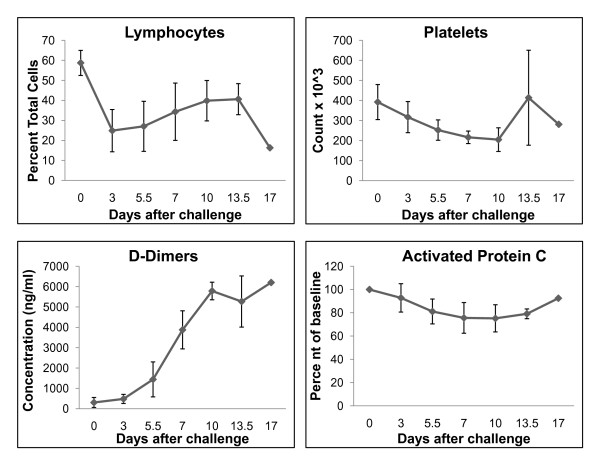
**Hematology and coagulation values after infection of cynomolgus monkeys with Lassa virus**. Development of lymphopenia (top left panel) and thromobocytopenia (top right panel). Increases in circulating levels of D-dimers (bottom left panel) and decreases in circulating levels of activated protein C (bottom right panel).

There was a pronounced lymphopenia as lymphocytes dropped from approximately 59% of the leukocyte population just prior to LASV challenge (day 0) to less than 25% on day 3, and then appeared to slightly rebound to approximately 40% by day 10 (Figure 1). Although still within normal reference range, circulating platelet numbers declined from a mean of 392 × 10*/mm* (range 100-700 × 10*/mm*) before LASV challenge (day 0) to 205 × 10*/mm* on day 10 (Figure 1). However, by day 13.5, platelet numbers returned to pre-challenge levels (mean 414 × 10*/mm*). Development of fibrin degradation products (D-dimers) showed increases of 12-fold by day 7 and near 20-fold by day 10 (Figure 1). Plasma levels of protein C remained unchanged at day 3 but decreased by approximately 25% at days 7 and 10 (Figure 1).

**Figure 2:**
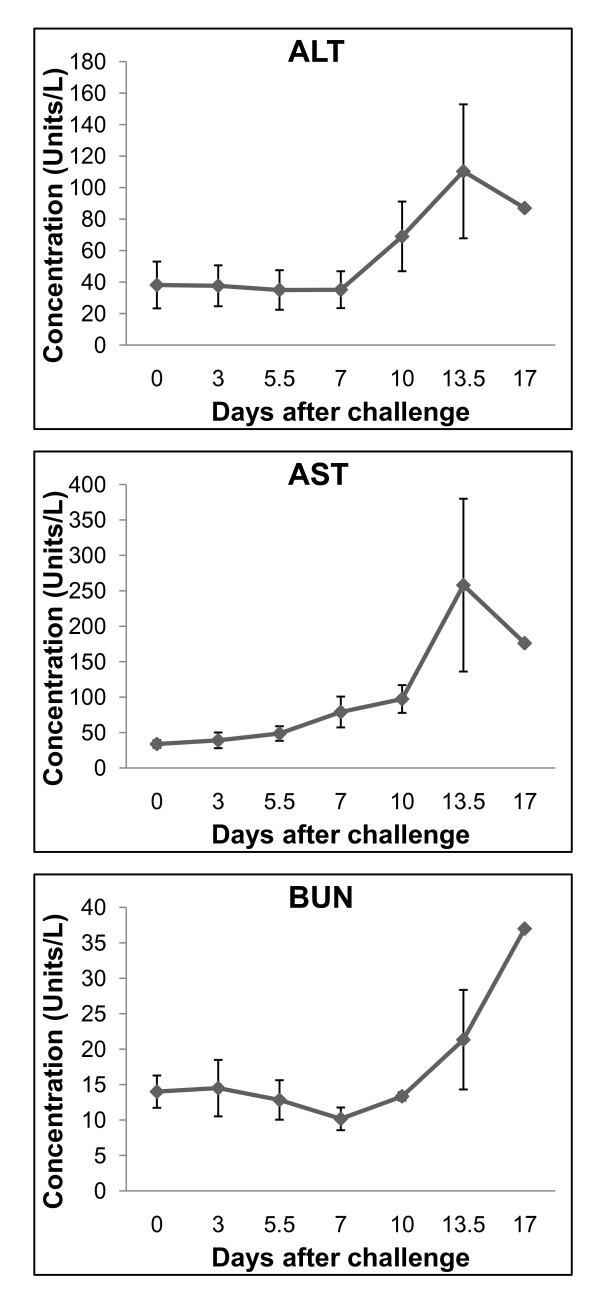
**Clinical chemistry values after infection of cynomolgus monkeys with Lassa virus**. Elevated levels of serum enzymes primarily at the late stages of disease (days 13-17). Top left panel, alanine aminotransferase (ALT). Top right panel, aspartate aminotransferase (AST). Bottom left panel, blood urea nitrogen (BUN).

Early serum enzyme levels were unremarkable, but many were elevated during the late stages of disease. On day 10, aspartate aminotransferase (AST) increased nearly 3-fold (mean, 97 IU/L) as did alanine aminotransferase (ALT) (mean, 69 IU/L) by day 13.5 (Figure 2). Blood urea nitrogen (BUN) levels remained generally within normal limits through day 10 and increased nearly 2-fold over baseline on day 13.5 (Figure 2). Serum albumin levels slightly decreased from a pre-LASV challenge mean of 3.0 g/dL to 1.8 g/dL by day 17; however, total protein levels did not substantially fluctuate during the course of infection.

### Necropsy findings

**Figure 3:**
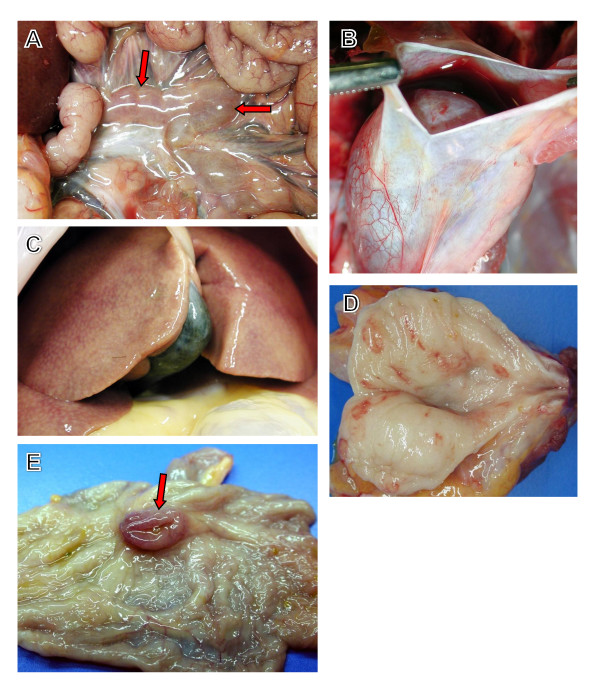
**Representative gross necropsy lesions from nonhuman primates experimentally infected with Lassa virus**. (*A*) Enlargement and moderate congestion of mesenteric lymph nodes (arrows) at day 15. (*B*) Accumulation of fluid in the pericardial cavity of a cynomolgus monkey 17 days after infection with Lassa virus. (*C*) Reticulation and discoloration of the liver 15 days after infection with Lassa virus. (*D*) Multifocal red foci that microscopically corresponded with inflammation on the mucosal surface of the urinary bladder at day 15. (*E*) Congestion/hemorrhage of the cecum occurring at day 15. The cecum is opened up and the ileum extends outward from the cecum; arrow indicates the ileocecal junction.

At day 7, axillary and inguinal lymphadenopathy and congestion were observed in all three animals (3/3) euthanized at this time point. The mandibular and mesenteric lymph nodes were also enlarged (3/3) and noticeable enlargement and congestion of the iliac lymph nodes was noted in one of the day 7 macaques. Additional findings at day 7 included friable spleens (1/3) and congestion at the ileocecal junction (1/3). By terminal time points (days 13.5-17), similar congestion and enlargement of the inguinal, axillary, mandibular, and mesenteric lymph nodes (Figure 3A) were also suggestive of LASV infection in all terminal animals (3/3). Other noteworthy findings at days 13.5-17 included: congested or pale yellow, friable livers (3/3) (Figure 3C); adrenal gland enlargement (1/3); pancreas enlargement (1/3); renal congestion (1/3); accumulation of red-tinged fluid in the pericardial sac (2/3) (Figure 3B); congestion at the ileocecal junction (1/3) (Figure 3E); and petechial hemorrhage on the mucosal surface of the urinary bladder (1/3) (Figure 3D).

### Virus titers in blood and tissues

**Figure 4:**
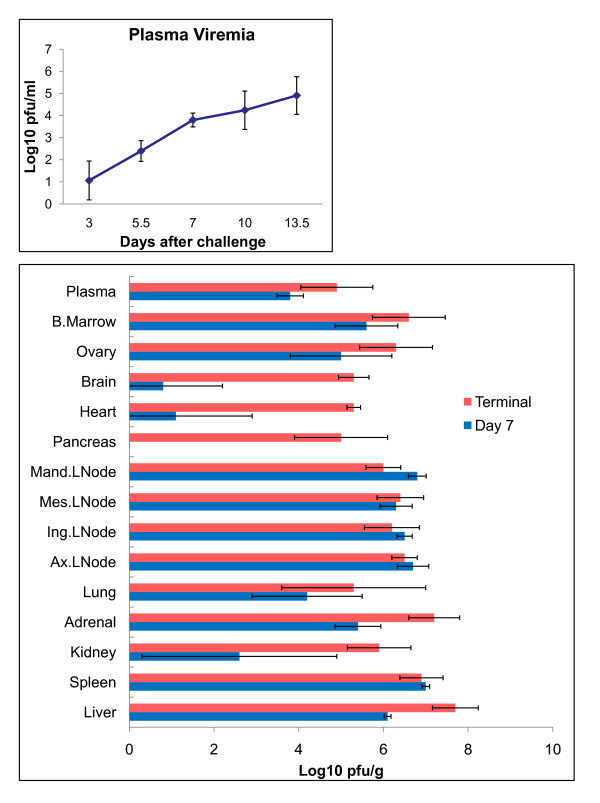
**Plasma and organ titers**. Mean Lassa infectivity of cynomolgus monkey plasma (top panel) and tissue homogenates (10% wt/vol) (bottom panel). LNode = lymph node.

The onset of plasma viremia occurred in four of six (4/6) animals on day 3 and in all animals (6/6) by day 5.5. Peak viremia (mean 5.0 log_10 _pfu/ml) occurred on day 13.5 (Figure 4). Infectious LASV was detected in all tissues except the pancreas of all day 7 animals (3/3) (Figure 4). The highest mean LASV titers at day 7 were detected in spleen and lymph nodes (6.3-7.0 log10 pfu/g) (Figure 4) suggesting that these organs are early sites of viral replication. By terminal time points (days 13.5-17) virus was detected in all tissues of all three terminal animals. The highest mean titers from these terminal animals were documented in the liver (7.7 log10 pfu/g), followed by adrenal gland (7.2 log10 pfu/g), and spleen (6.9 log10 pfu/g) (Figure 4).

### Analysis of cytokines/chemokines in circulation

**Figure 5:**
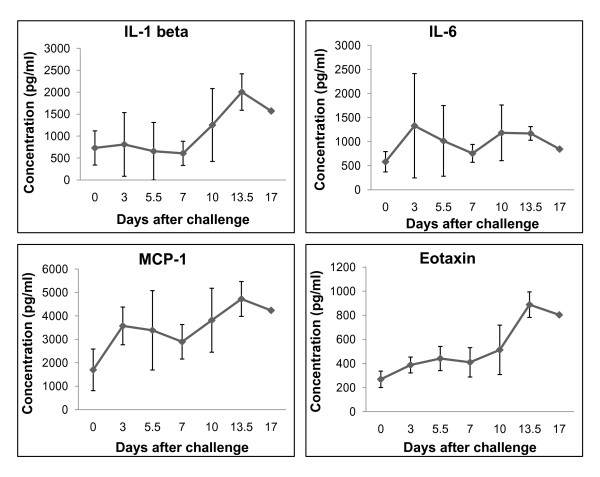
**Cytokines and chemokines**. Analysis of cytokine and chemokine accumulation in serum/plasma Lassa virus-infected cynomolgus monkeys.

In order to evaluate the immune response to LASV infection, plasma and/or sera were analyzed for levels of cytokines/chemokines. Significant increases (p < 0.05) in levels of MCP-1 were detected at days 3, 5.5, 7, 10, and 13.5 while significant increases in levels of eotaxin were observed at days 3, 7, and 13.5 after LASV challenge (Figure 5). Significant increases in levels of IL-6 and IL-1β were detected at day 13.5 (Figure 5). Increased levels of IFN-α, IFN-γ, IL-1R, IL-2R, IL-4, IL-5, IL-7, IL-8, IL-10, IL-12 p40/p70, IL-13, IL-15, IL-17, IP-10, GM-CSF, MIG, RANTES, and TNF-α were not detected in any animal at any time point.

### Histology and immunohistochemistry

Temporal results of LASV-infected cells demonstrated in primary target tissues by immunohistochemistry are shown in Table 2.

**Table 2:** Immunohistochemical findings from cynomolgus monkeys infected with Lassa virus

**Animal No**.	Days PI	Lymph nodes				Spleen			
		***Mono***	***Mac***	***Dend***	***End***	***Mono***	***Mac***	***Dend***	***End***

Subj 1	7	+	+	++	+	+	+	++	-

Subj 2	7	+	+	+++	+	+	+	+++	-

Subj 3	7	+	+	++	+	+	+	++	-

Subj 4	13	+	+	+	++	+	+	+	++

Subj 5	15	+	+	+	++	+	+	+	++

Subj 6	17	+	+	+	++	+	+	+	++

**Animal No**.	**Days PI**	**Liver/Adrenal**				**Brain**			

		***Kup***	***Hep***	***Cort***	***End***	***Neur***	***Glial***	***Mac***	***End***

Subj 1	7	-	+	+	-	-	-	-	+

Subj 2	7	++	++	++	-	-	-	+	+

Subj 3	7	+	+	+	-	-	-	+	-

Subj 4	13	++	++	+++	++	-	-	++	++

Subj 5	15	++	+++	+++	++	-	-	++	++

Subj 6	17	++	++	+++	++	-	+	++	++

+ indicates that LASV antigen-positive cells were rarely detected; + + indicates that LASV-positive cells were occasionally detected; + + + indicates that LASV-positive cells were frequently detected; and - indicates that no LASV-positive cells were detected.

*Mono *= monocytes; *Mac *= macrophages; *DC *= dendritic cells; *End *= Endothelial cells; *Kup *= Kupffer cells; *Hep *= hepatocytes; *Cort *= adrenal cortical cells; *Neur *= Neurons.

#### Lymphoid tissues

##### Lymph nodes

**Figure 6:**
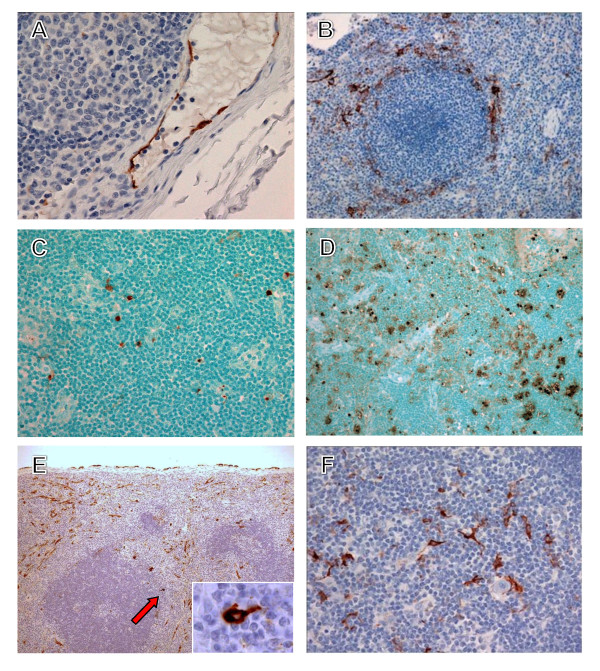
**Immunohistochemistry and TUNEL staining in lymphoid tissues of cynomolgus monkeys**. (*A*) Immunopositive endothelial cells (brown) in an axillary lymph node at day 13. (*B*) Immunopositive tissue macrophages and dendritic cells (brown) in spleen at day 7. (*C*) TUNEL-positive (brown) lymphocytes and scattered tingible body macrophages in an axillary lymph node at day 7. (*D*) TUNEL-positive (brown) lymphocytes and scattered tingible body macrophages in an axillary lymph node at day 17. (*E*) Immunopositive tissue macrophages, endothelial cells, and dendritic cells (brown) in spleen at day 13. Inset is enlargement of indicated area (arrow) showing dendritic cell. (*F*) Immunopositive dendritic cells (brown) in thymus at day 7. Original magnifications, ×40 (*A*), ×20 (*C*, *F*), ×10 (*B*, *D*, *F*).

LASV antigen was frequently detected in dendritic cells of all lymph nodes of all animals (3/3) at day 7 and rarely detected in endothelial cells of 2/3 animals at day 7. Sinus histiocytosis and mild lymphocytolysis was seen in some, but not all lymph nodes in the three animals euthanized at this time point. By terminal time points (days 13.5-17) there was less extensive immunopositive staining of dendritic cells in all lymph nodes of all three animals. Increased numbers of immunopositive endothelial cells were detected in all lymph nodes of all terminal animals (3/3) (Figure 6A). An increase in TUNEL-positive lymphocytes and scattered tingible body macrophages with cytoplasmic TUNEL-positive debris was observed in the axillary lymph node of 1/3 terminal animals (Figure 6D) when compared with day 7 animals (Figure 6C). However, TUNEL results from inguinal and mesenteric lymph nodes were inconclusive.

##### Spleen

**Figure 7:**
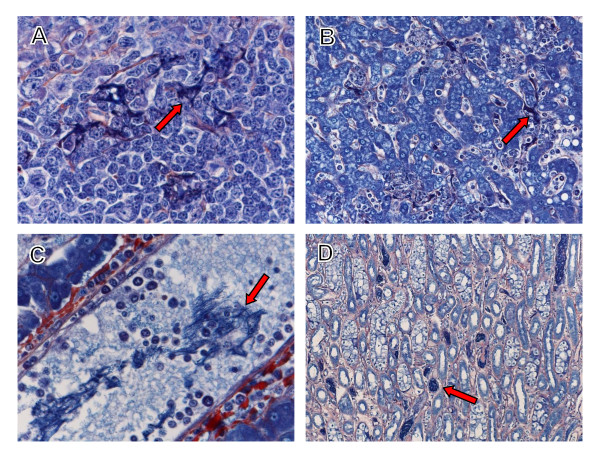
**PTAH staining of Lassa virus-infected cynomolgus monkey tissues**. Polymerized fibrin (arrows) in the marginal zone of spleen (*A*), sinusoids (*B*) and vessels (*C*) in liver, and medullary vessels of the kidney (*D*) at day 15. Original magnifications, ×10 (*D*), ×20 (*B*), ×40 (*A*), ×60 (*C*).

At day 7, LASV immunostaining was noted in cells morphologically consistent with dendritic cells primarily in the marginal zone (Figure 6B) and to a lesser extent in monocytes and tissue macrophages in the marginal zones and red pulp. Mild lymphocytolysis was noted in 2/3 animals. PTAH staining demonstrated infrequent deposition of polymerized fibrin in the red pulp and marginal zone of 1/3 animals at this time point. By terminal time points (days 13.5-17) LASV immunopositive dendritic cells were detected in spleen of all animals (3/3) (Figure 6E) but were less frequently observed than in day 7 animals. Increased numbers of immunopositive endothelial cells were detected in spleen of all terminal animals (3/3). Splenic lymphocytes were consistently LASV immunonegative in all animals at all time points (6/6). There appeared to be a slight increase in TUNEL-positive lymphocytes and scattered tingible body macrophages with cytoplasmic TUNEL-positive debris in the terminal animals (3/3). Detection of polymerized fibrin by PTAH staining was occasionally present in the red pulp and marginal zones of terminal animals (3/3) (Figure 7A) with deposits being most prominent in the animal that succumbed on day 15.

##### Thymus

At day 7, LASV antigen distribution and pathology was similar to that described for lymph nodes and spleen with frequent immunostaining of dendritic cells (Figure 6F). By terminal time points marked atrophy was apparent in all three animals (3/3).

##### Liver

**Figure 8:**
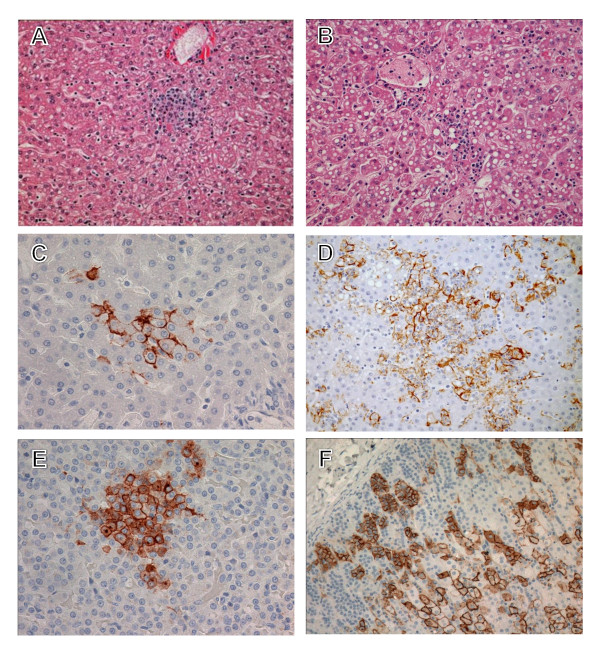
**Histopathology and immunohistochemistry liver and adrenal gland of Lassa virus-infected cynomolgus monkeys**. Histology of liver showing inflammation at days 7 (*A*) and 15 (*B*); H&E stain. Note the progression of increased immunostaining (brown) of hepatocytes (*C*) and adrenal cortical cells (*E*) from day 7 to day 15 (*D*, liver; *F*, adrenal gland). Original magnifications, ×10 (*A*, *B, D, F*), ×20 (*C, E*).

Histologically, the severity of hepatic changes varied between animals at day 7. The most noteworthy lesions were lymphoplasmacytic and neutrophilic inflammation (2/3) (Figure 8A). LASV antigen was detected in Kupffer cells and hepatocytes (3/3) at day 7 (Figure 8C). Lymphoplasmacytic and neutrophilic inflammation was more pronounced in the terminal animals (3/3) (Figure 8B). LASV immunostaining increased substantially at terminal time points (Figure 8D) with increased numbers of immunopositive hepatocytes (3/3). PTAH staining demonstrated multifocal distribution of small fibrin deposits in sinusoids (Figure 7B) and vessels (Figure 7C).

##### Endocrine system

No significant lesions were detected in the adrenal glands or pancreas at day 7. However, small foci of LASV-positive adrenal cortical cells were detected in the zona glomerulosa and zona fasciculata of 2/3 animals (Figure 8E). By terminal time points there was multifocal necrosis of adrenal cortical cells in all animals (3/3). LASV immunostaining increased substantially at terminal time points (Figure 8F) with increased numbers of immunopositive adrenal cortical cells in all zones (3/3). In addition, LASV antigen was occasionally detected in pancreatic islets of 2/3 terminal macaques.

##### Reproductive organs

At day 7, histiocytic inflammation was noted in ovaries and uterus of 1/3 animals. LASV antigen was associated with histiocytes in these inflammatory foci. In addition, there was infrequent LASV immunoreactivity of thecal cells surrounding ovarian follicles in 2/3 animals at this time point. By terminal time points, lymphoplasmacytic and histiocytic inflammation was a prominent finding in the ovaries of 2/3 animals immunoreactivity of granulosa cells. LASV antigen was associated with histiocytes in these inflammatory foci and granulosa cells near these areas of inflammation. Lymphocytic and histiocytic inflammation was noted in the uterus and there was multifocal strong immunoreactivity for LASV in uterine endothelial cells (3/3).

##### Kidneys and urinary bladder

No significant renal or bladder lesions were observed at day 7. Likewise, LASV was rarely detected in interstitial and medullary capillaries at this time point (1/3). At terminal time points, lymphoplasmacytic and neutrophilic inflammation was observed in multiple foci of kidney of all animals (3/3). LASV-positive endothelial cells were randomly scattered throughout the kidney and surrounding connective tissue (3/3). Multifocal lymphoplasmacytic and histiocytic inflammation were noted in the bladder of 2/3 terminal animals. Immunopositive transitional epithelial cells, fibroblasts, and endothelial cells were also detected in the bladder of 2/3 terminal animals. PTAH-positive fibrin was multifocally detected in medullary vessels in kidney of all terminal animals (3/3) (Figure 7D) with deposits being most prominent in the animal that succumbed on day 15.

##### Lung

No significant pulmonary lesions were noted at day 7. At terminal time points one animal (1/3) had mild interstitial pneumonia. LASV antigen was detected multifocally in endothelial cells (3/3) and macrophages (1/3).

##### Heart

Lesions at day 7 included histiocytic and neutrophilic inflammation of the myocardium (1/3). LASV antigen was detected in histiocytic inflammatory cells of this animal. By the terminal time points, lesions were more prominent and included plasmacytic and histiocytic inflammation of the myocardium (3/3) and necrotizing coronary arteritis (1/3). LASV antigen was detected in histiocytic inflammatory cells (3/3) and vascular smooth muscle cells (1/3) at this time point.

##### Nervous system

**Figure 9:**
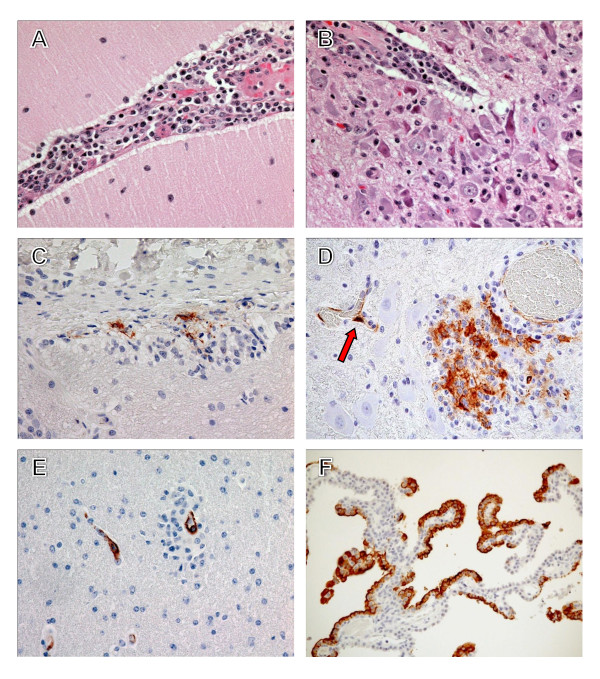
**Histopathology and immunohistochemistry of nervous tissue of Lassa virus-infected cynomolgus monkeys**. (*A*) Histology of cerebellum showing meningitis at day 15; H&E stain. (*B*) Histology of cerebrum showing neuronal necrosis at day 13; H&E stain. (*C*), Optic neuritis; note immunopositive inflammatory foci (brown) at day 15. (*D*), Brain stem, immunopositive endothelium (arrow) and immunopositive inflammatory foci (brown) in the neuropil peripheral to a medium-sized blood vessel at day 13. (*E*) Immunopositive endothelial cells (brown) in cerebrum at day 15. (*F*) Choroid plexus, prominent immunostaining (brown) of the cuboidal epithelial cells lining the choroid at day 15. Original magnifications, ×20 (*B-F*), ×40 (*A*)

No significant lesions were observed in the nervous system at day 7. However, LASV immunostaining was infrequently detected in circulating histiocytic cells (2/3) and in endothelial cells (2/3) at this timepoint. Substantial changes in lesions and the presence of LASV occurred in the neuropil by terminal time points. Noteworthy findings in terminal animals included: meningoencephalitis in the cerebrum, cerebellum (Figure 9A), and brainstem (3/3); neuronal necrosis and gliosis in cerebrum (Figure 9B) and brainstem (3/3); choroid plexitis (1/3); meningomyelitis (1/3); and neuritis of optic nerve (2/3) (Figure 9C). LASV antigen was detected multifocally in endothelial cells and histiocytic inflammatory cells of cerebrum (Figures 9D,E), cerebellum, brainstem, spinal cord, and optic nerve (Figure 9C) of terminal animals. In addition, LASV antigen was detected in glial cells in cerebrum of 2/3 animals and in choroid plexis epithelial cells in cerebrum of 1/3 animals (Figure 9F).

## Discussion

Despite an estimated 3,000 fatal cases of LF per year in West Africa [1], there have been relatively few postmortem histologic or immunohistochemical studies [14,36-43]. Morphologic features from these cases include hepatic and adrenal necrosis, splenic necrosis centered in the marginal zone, mild myocarditis, mild interstitial pneumonia, and lymphoid depletion. Interestingly, the extent of lesions observed in any of these tissues is insufficient to account for death. Histologic evaluation of terminal tissues from LASV-infected macaques in the current study and previous studies [19,20,34] mirrored these observations from human cases. In addition, LASV infection of cynomolgus macaques in the current study produced multifocal to severe neuropathology at terminal stages of disease that was characterized by central nervous system lesions associated with LASV antigen. These findings are consistent with neurologic complications described in human LF including confusion, tremor, seizure, convulsion, and coma which occur frequently in critically ill patients who often succumb after the onset of these symptoms [40,44,45].

Coagulations disorders, which are hallmark features of viral HF do not appear to be as prominent in the majority of cases of human LF [14,26,41]. For example, while thrombocytopenia appears to be a consistent finding among HF virus infections of humans and nonhuman primates it is not a prominent feature of LASV infection of humans or nonhuman primates [14,22,23,26,41]. Moderate thrombocytopenia was reported in patients with severe LF, but the most significant changes were noted in platelet function which was markedly depressed in patients and in LASV-infected nonhuman primates [24,26]. Overall, increased circulating levels of D-dimers and slight decreases in plasma levels of protein C were noted in the LASV-infected macaques in the current study but these changes were modest compared to changes reported in macaques infected with other HF viruses such as Ebola [35]. However, hemorrhage has been reported in a subset of LF patients and is associated with high mortality [4,25,26]. Consistent with human LF, LASV infection of nonhuman primates appears to produce different degrees of coagulopathy in individual animals with some animals showing more severe coagulation disorders than others.

LASV infection of cynomolgus monkeys was characterized by a dysregulation of the host immune response. In this study, we observed increased circulating levels of proinflammatory cytokines/chemokines including IL-1β, IL-6, MCP-1, and eotaxin. These results are consistent with a previous study of LASV infection of macaques which associated elevated levels of circulating IL-6 with poor outcome [34]. In addition, our results for circulating levels of cytokines/chemokines in LASV-infected macaques were consistent with limited investigations of LASV-infected patients. Specifically, Mahanty et al. showed high levels of circulating IL-6 in fatal human cases of LF [46]. These investigators also reported that nonfatal LF is associated with high levels of circulating IL-8 and IP-10. Consistent with these findings we failed to detect any increases in levels of IL-8 and IP-10 in moribund LASV-infected cynomolgus monkeys.

A primary goal of the current study was to begin to characterize the pathogenesis of LASV infection at times before death. Evaluation of tissues by conventional histology and immunohistochemistry demonstrated a consistent pattern of LASV infection in cynomolgus monkeys starting with dendritic cells in the lymphoid tissues progressing to infection of Kupffer cells in liver and parenchymal cells in liver and adrenal gland, endothelial cells in a variety of tissues including nervous tissue, and finally to infection of the epithelium. The prominent infection of dendritic cells in all three LASV-infected cynomolgus macaques at day 7 is a particularly important finding. Dendritic cells were also shown to be early targets of other HF viruses in nonhuman primates [47]. Dendritic cells function as sentinels of the adaptive immune system; they are located in the peripheral tissues where they capture and process exogenous antigens and migrate to regional lymph nodes where they undergo maturation. Early infection of dendritic cells likely plays a key role in the immunosuppression that is a hallmark feature of LF in primates. Our *in vivo *findings are consistent with previous *in vitro *studies demonstrating that LASV infection of dendritic cells impairs their function and fails to activate the cells [48,49].

The sequence of morphological, cytologic, virologic, serological, and inflammatory change following LASV in cynomolgus monkeys creates a useful model in the study of LF and provides a basic understanding of the disease model and pathogenesis. This understanding is critical for characterizing the LF nonhuman primate model, identifying gaps in knowledge as well as identifying critical pathways for validation with limited available human data. This understanding and validation are critical for the advancement of any prophylactic or therapeutic product through the FDA under the Animal Efficacy Rule.

## Ethics

Research was conducted in compliance with the Animal Welfare Act and other federal statutes and regulations relating to animals and experiments involving animals and adheres to principles stated in the *Guide for the Care and Use of Laboratory Animals*, National Research Council, 1996. The facility where this research was conducted is fully accredited by the Association for Assessment and Accreditation of Laboratory Animal Care International.

## Competing interests

The authors declare that they have no competing interests.

## Authors' contributions

TWG and LEH conceived and designed the experiments. TWG wrote the animal protocol for the studies. TWG, JBG, and KMDD performed the Lassa challenge experiments in nonhuman primates. JBG and KMDD performed the clinical pathology assays. LEH performed the Bio-Plex and coagulation assays. JBG performed the Lassa virus infectivity assays. MAS and TL performed the pathology studies. LEH, MAS, JBG, EAF, KMDD, TL, and TWG analyzed the data. TWG wrote the paper. LEH, MAS, EAF, and TL edited the manuscript.
